# Liproxstatin-1 attenuates acute hypertriglyceridemic pancreatitis through inhibiting ferroptosis in rats

**DOI:** 10.1038/s41598-024-60159-7

**Published:** 2024-04-25

**Authors:** Xuelian Xiang, Mengtao Xu, Li Liu, Nuo Meng, Yu Lei, Yong Feng, Guodu Tang

**Affiliations:** Department of Gastroenterology, The First Affiliated Hospital of Guangxi Medical University, No. 6 Shuangyong Road, Nanning, 530021 China

**Keywords:** Endocrine system and metabolic diseases, Gastrointestinal diseases, Diseases, Metabolism, Acute inflammation

## Abstract

Ferroptosis is closely associated with inflammatory diseases, including acute pancreatitis (AP); however, the involvement of ferroptosis in hypertriglyceridemic pancreatitis (HTGP) remains unclear. In the present study, we aimed to explore the relationship between lipid metabolism and ferroptosis in HTGP and the alleviating effect of liproxstatin-1 (Lip-1) in vivo. This study represents the first exploration of lipid metabolism and endoplasmic reticulum stress (ERS) in HTGP, targeting ferroptosis as a key factor in HTGP. Hypertriglyceridemia (HTG) was induced under high-fat diet conditions. Cerulein was then injected to establish AP and HTGP models. Lip-1, a specific ferroptosis inhibitor, was administered before the induction of AP and HTGP in rats, respectively. Serum triglyceride, amylase, inflammatory factors, pathological and ultrastructural structures, lipid peroxidation, and iron overload indicators related to ferroptosis were tested. Moreover, the interaction between ferroptosis and ERS was assessed. We found HTG can exacerbate the development of AP, with an increased inflammatory response and intensified ferroptosis process. Lip-1 treatment can attenuate pancreatic injury by inhibiting ferroptosis through lipid metabolism and further resisting activations of ERS-related proteins. Totally, our results proved lipid metabolism can promote ferroptosis in HTGP by regulating ACSL4/LPCAT3 protein levels. Additionally, ERS may participate in ferroptosis via the Bip/p-EIF2α/CHOP pathway, followed by the alleviating effect of Lip-1 in the rat model.

## Introduction

Acute pancreatitis (AP) is a leading cause of gastrointestinal-related hospitalizations in the United States^1^. Globally, there has been a significant increase in the morbidity and mortality associated with AP^2^. Major etiologies of AP include gallstones, alcohol abuse, and hypertriglyceridemia (HTG). Studies indicate a higher prevalence of HTG-induced acute pancreatitis (HTGP) in Asia compared to Western countries^3,4^. Jin et al. reported a rise in the proportion of HTGP from 14% in 2001 to 34% in 2016, ranking second to gallstone-related AP^5^. Patients with HTGP exhibit a poorer prognosis and more severe complications compared to other etiologies^6^. To date, the mechanism of HTGP remains controversial.

Ferroptosis, a novel mode of cell death driven by iron-dependent lipid peroxidation, was coined by Dixon in 2012^7^. It induces cell death by upregulating reactive oxygen species (ROS) levels and disrupting redox balance^8^. Morphologically, ferroptosis is characterized by shrunken mitochondria, decreased crista, and condensed mitochondrial membrane^7,9^. Over the past decade, evidence has linked ferroptosis to various diseases, including cancer, ischemia–reperfusion injury, and AP^10–12^. While programmed cell death such as apoptosis, autophagy, pyroptosis, and necrosis has been associated with HTGP onset^13–16^, the involvement of ferroptosis in HTGP pathogenesis remains largely unexplored.

Studies have identified key regulators in ferroptosis, including glutathione peroxidase 4 (GPX4), cysteine/glutamate transporter (xCT), acyl-CoA synthetase long-chain family member 4 (ACSL4), and lysophosphatidylcholine acyltransferase 3 (LPCAT3). GPX4 and xCT play crucial roles in converting toxic lipid hydroperoxides to non-toxic lipid alcohols through glutathione (GSH) synthesis^10^, thereby protecting cells from lipid peroxidation and ROS injury. Inhibition or knockdown of *GPX4* can contribute to ferroptosis in vivo^17,18^. ACSL4 and LPCAT3, key enzymes in polyunsaturated fatty acids (PUFAs) metabolism, have recently been identified as essential in regulating lipid composition in ferroptosis^19^. Furthermore, anomalous expression of ACSL4 can induce neuronal death through ferroptosis, whereas knockdown of *ACSL4* has been shown to alleviate brain injury^20^. However, the role of ACSL4 in HTGP progression is yet unclear, and there is currently no research focusing on the relationship between HTGP and ACSL4 or LPCAT3.

The endoplasmic reticulum (ER) is responsible for protein synthesis, folding, and maturation in cells^21^. Harmful stimuli can induce unfolded protein response and ER stress (ERS) to maintain cellular homeostasis^22^. Li et al. revealed that ERS can trigger pancreatitis as pancreatic acinar cells participate in protein production^23^. However, little is known about the interaction between ferroptosis and ERS in the prognosis of HTGP.

Crucially, the relationship between ferroptosis and ERS in HTGP has been rarely explored. In this study, an HTGP model in rats was established to further investigate the role of ferroptosis in HTGP. A high-fat diet was observed to exacerbate the severity of AP through ERS, inducing ferroptosis. This study demonstrates elevated levels of lipid metabolism-associated proteins, ACSL4 and LPCAT3, which can trigger the progression of ferroptosis. Therefore, regulating lipid metabolism may offer a new perspective in the treatment of HTGP through ferroptosis. In conclusion, ferroptosis may be a potential treatment target for HTGP, warranting further investigation in the long run.

## Materials and methods

### Animals and models

**Figure 1:**
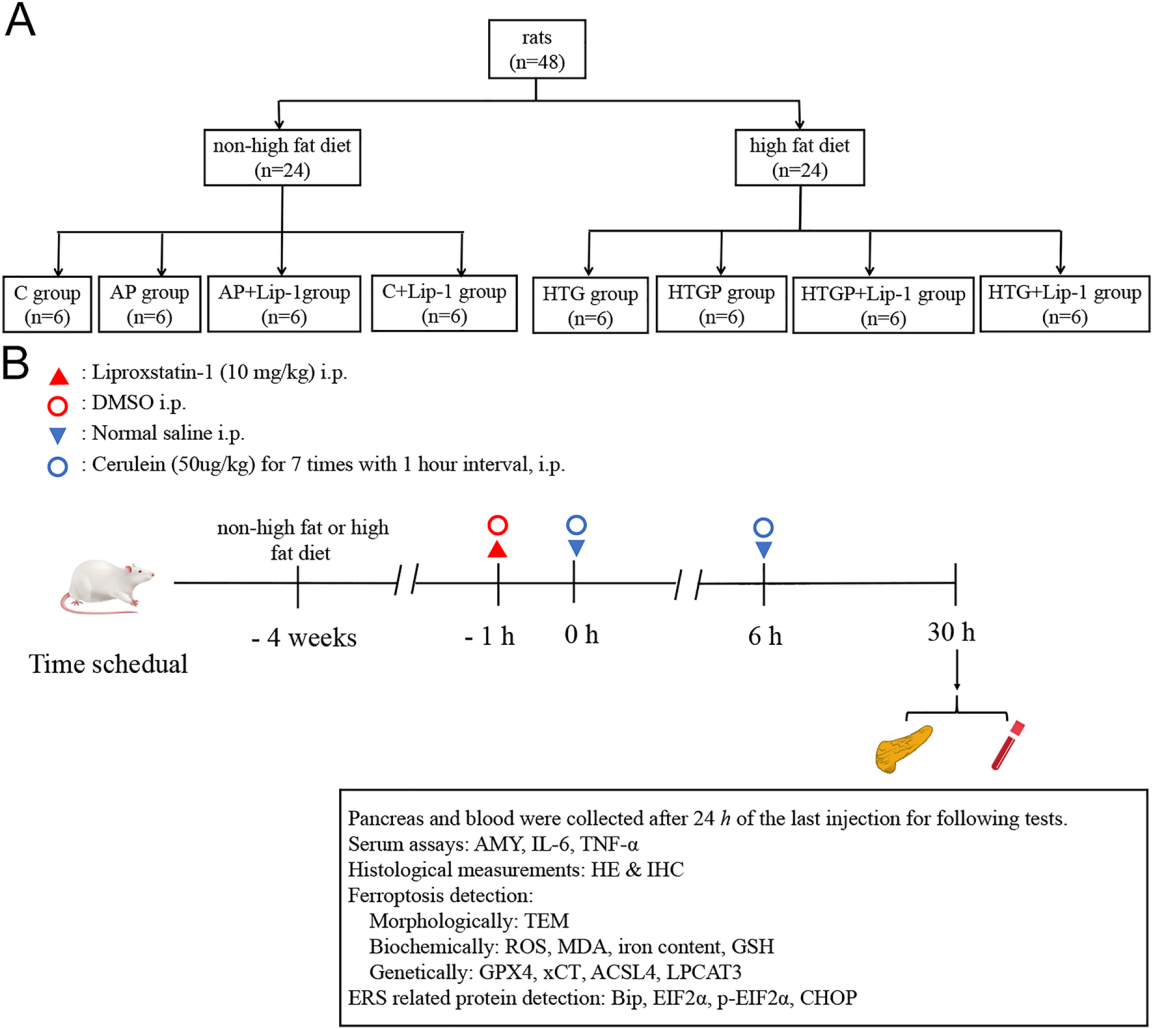
The procedure of rat model and treatment time (*n* = 6). (**A**) Animal modeling group and (**B**) treatment schedule of rats. C, control; AP, acute pancreatitis group; Lip-1, liproxstatin-1; HTG, hypertriglyceridemia; AMY, amylase; TEM, transmission electron microscopy; ROS, reactive oxygen species; MDA, malondialdehyde; GSH, glutathione; GPX4, glutathione peroxidase 4; xCT, cysteine/glutamate transporter; ACSL4, acyl-CoA synthetase long-chain family member 4; LPCAT3, lysophosphatidylcholine acyltransferase.

The study was approved by the Ethics Committee of the First Affiliated Hospital of Guangxi Medical University (No. 2023-E666-01) and conducted in accordance with the regulatory guidelines. All methods were reported in accordance with ARRIVE guidelines. In this present study, 48 Sprague–Dawley (SD) male rats (70–80 g, 4 weeks) were obtained from the Laboratory Animal Center of Guangxi Medical University and housed in standard conditions (a 12/12 h light/dark cycle, 25 °C temperature, and 60% humidity). All rats had ad libitum access to food and water. The HTG rats were fed with high-fat diet (82.8% normal diet, 15% lard, 2% cholesterol, 0.2% sodium cholate) based on previous studies^24^, whereas rats in another group were treated with only a normal diet. After 4 weeks of feeding, the rats were randomly assigned to eight groups, each consisting of six rats: non-HTG control (C group), AP group, AP + Lip-1 group, control + Lip-1 (C + Lip-1 group), HTG-control (HTG group), HTG-induced AP (HTGP group), HTGP + Lip-1 group, and HTG + Lip-1 group (Fig. 1A). The rats were fasted overnight before the experiment. AP and HTGP models were established by seven intraperitoneal injections of cerulein (50 μg/kg) with 1 h interval, whereas rats in the other groups received normal saline injections. Lip-1 pretreatment (10 mg/kg) was administered 1 h before AP and HTGP models. All rats were treated with the same volume of vectors. All rats were alive after 24 h of the last injection and were sacrificed by CO_2_ asphyxiation (Fig. 1B).

### Specimen collection

Blood was collected by abdominal aortic puncture, and serum was isolated using a precooled centrifuge (4 °C, 3500 rpm for 10 min) and preserved at –20 °C until analysis. Fresh pancreas tissue was excised and divided into two tubes; a portion of the pancreas was fixed in 4% paraformaldehyde for pathological experiments, while the remaining tissue was preserved at – 80 °C for later investigation.

### Serum assay

The levels of serum amylase (AMY) and triglycerides (TG) were tested using an automatic biochemical analyzer in the clinical laboratory of the First Affiliated Hospital of Guangxi Medical University (Nanning, China). Interleukin-6 (IL-6, Fankew, Shanghai Kexing Trading Co, Ltd, China) and tumor necrosis factor-alpha (TNF-α, Fankew, Shanghai Kexing Trading Co, Ltd, China) levels were measured by ELISA kits following the manufacturer’s instructions.

### Lipid peroxidation and iron detection

Briefly, the content of GSH, malondialdehyde (MDA), and iron in the pancreas was measured by a GSH detection kit (Jiancheng, Nanjing, China), an MDA assay kit (Solarbio, Beijing, China), and an iron assay kit (Jiancheng), respectively, according to the manufacturer's instructions.

### ROS test assay

After diminished autofluorescence by AutoFluo quencher, frozen sections of pancreatic tissue were incubated in fluorescence probe for ROS (Sigma-Aldrich, St. Louis, MO, USA) under controlled conditions (30 min, avoiding light). Subsequently, the cell nuclei of the sections were stained with diamidinophenylindole (Servicebio, Wuhan, China). The ROS levels were measured by fluorescence microscope.

### Transmission electron microscopy (TEM)

Fresh pancreas tissues were pre-fixed in phosphate-buffered saline containing 2.5% glutaraldehyde at 4 °C overnight immediately after isolation from rats. The tissues were then post-fixed in 1% osmium tetroxide for 2 h at 25 °C. Following dehydration in ethanol and embedded in epoxy resin, the tissues were cut into 1 mm^3^ particles through ultramicrotome and stained with uranyl acetate. Moreover, ultrastructural images of the pancreas were acquired by Hitachi 7600 TEM (Tokyo, Japan).

### Histopathological measurement

Pancreas tissues were fixed in formalin, embedded in paraffin, and sliced (5 μm). Hematoxylin and eosin (H&E) staining were performed on the samples. For immunohistochemistry (IHC) analysis, the sections were incubated with primary antibodies against GPX4 (1: 200; BOSTER, Wuhan, China), ACSL4 (1:200; Sangong biotech, Shanghai, China), and LPCAT3 (1:200; Abcam) at 4 °C overnight. The sections were then exposed to horseradish peroxidase secondary antibodies (1:200; Servicebio) for 30 min at 25 °C. The section images were captured under optical microscopy, and the results were analyzed separately based on the experimental groups.

### Western blotting

Total protein from pancreatic tissue was obtained by RIPA lysis buffer with 1% phenylmethylsulfonylfluoride (Solarbio). The proteins were electrophoresed on 10% and 12% SDS-PAGE gels and transferred onto polyvinylidene fluoride membranes (Millipore, Darmstadt, Germany). After being blocked in 5% skimmed milk for 2 h at 25 °C, the membranes were cut prior to hybridisation with primary antibodies. After that, the membranes were incubated at 4 °C overnight with primary antibodies: GPX4 (1:1000; BOSTER), xCT (1:1000; Abcam), ACSL4 (1:1000; Abcam), LPCAT3 (1:500; ABclonal, Wuhan, China), binding immunoglobulin protein (Bip, 1:1000; Zenbio, Chengdu, China), C-EBP homologous protein (CHOP, 1:1000; BOSTER, Wuhan, China), eukaryotic initiation factor 2α (EIF2α, 1:2000; Proteintech, Wuhan, China), phosphoration of EIF2α (p-EIF2α, 1:1000; Abcam), and β-actin (1:1000; Cell Signaling Technology, Danvers, MA, USA). Subsequently, the membranes were incubated with a secondary goat anti-rabbit antibody (1:10,000; Cell Signaling Technology) for 2 h at 25 °C. Protein bands were visualized using the Odyssey Fc Imaging System, and band images were analyzed using Image J software. All initial western blotting bands in this study are presented in the Supplementary Information.

### Statistical analysis

Data analysis was performed using SPSS 25.0 software (IBM, Armonk, NY, USA). All data were expressed as mean ± standard deviation (SD) and visualized using GraphPad Prism 6.0. Experiments were repeated at least twice for result consistency. A *P*-value < 0.05 was considered statistically significant.

### Ethical declarations

The animal study protocol was approved by the Ethics Committee of the First Affiliated Hospital of Guangxi Medical University (2023-E666-01).

## Results

### Successful establishment of the rat model with cerulein and high-fat feeding

**Figure 2:**
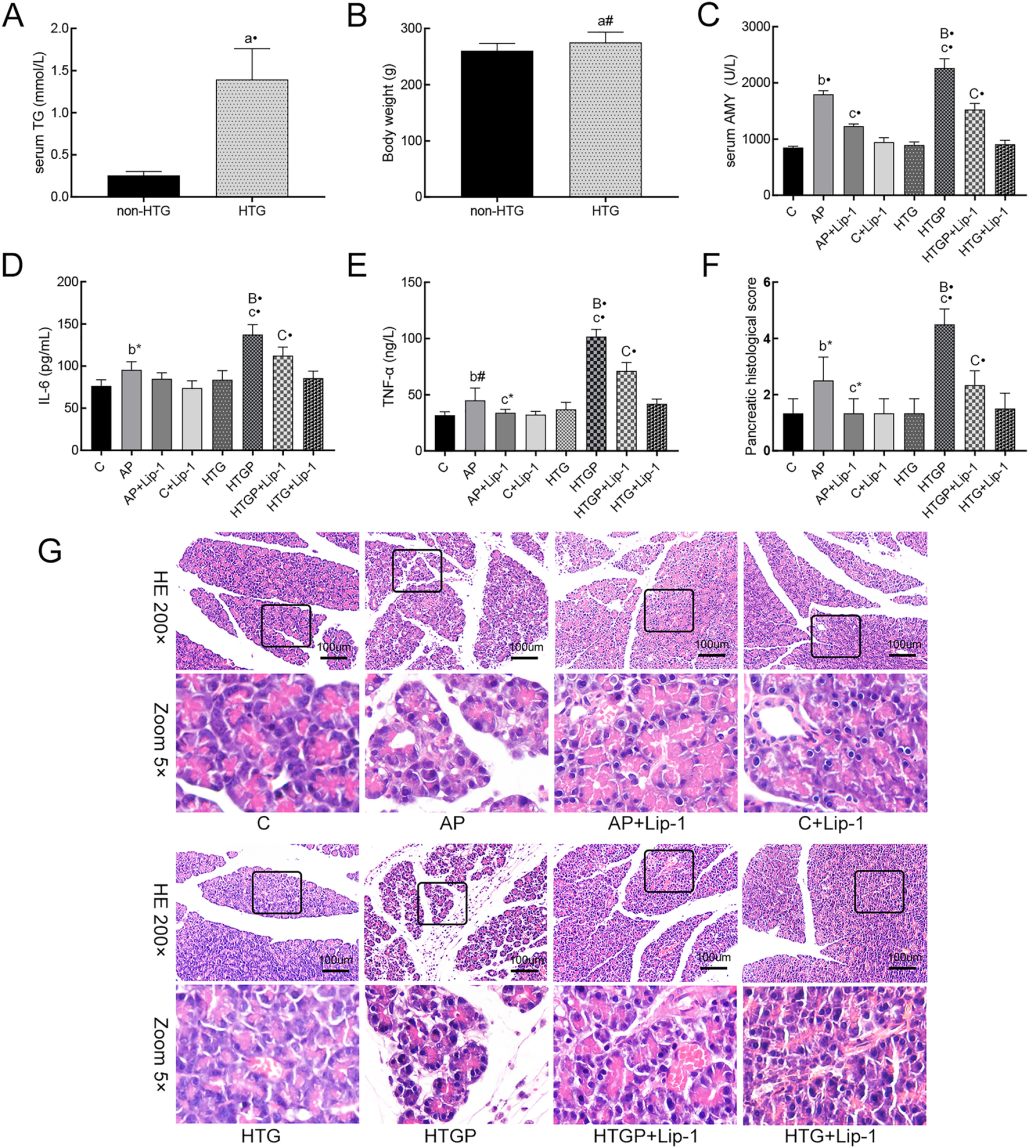
Successful establishment of AP and HTGP in vitro (*n* = 6). (**A**) Serum levels of TG; (**B**) Body weight of the rats; (**C**) Serum levels of AMY; (**D**) Serum levels of IL-6; (**E**) Serum levels of TNF-α in rats; (**F**) Pancreatic histological scores in rats; (**G**) Representative pancreas H&E stained tissue under microscope (×200); TG, triglyceride; AMY, amylase; C, control; AP, acute pancreatitis; HTG, hypertriglyceridemic; HTGP, HTG pancreatitis; *a vs. the non-HTG group, *b vs. the C group, *c vs. the AP group, *B vs. the HTG group, *C vs. the HTGP group, *P < 0.05, ^#^*P* < 0.01, •*P* < 0.001.

The serum TG in the HTG group was significantly higher than that in the non-HTG group presented in Fig. 2A (0.26 ± 0.04 vs. 1.39 ± 0.37, *P* < 0.001). As presented in Fig. 2B, the body weight in the non-HTG group was slightly lower than that in the HTG group (260.60 ± 12.92 vs. 275.30 ± 18.13, *P* < 0.01). As depicted in Fig. 2C–E, the levels of AMY, IL-6, and TNF-α in AP and HTGP groups were increased compared to those in the corresponding control and HTG groups, with levels in the HTGP group significantly higher than those in the AP group. Furthermore, Lip-1 pretreatment resulted in a reduction in the levels of AMY, IL-6, and TNF-α. Notably, the levels of IL-6 and TNF-α were significantly higher in the HTGP group than those in the AP group. Additionally, the histological microscopic view of the pancreas in rats (Fig. 2G) revealed leukocyte infiltration and interstitial edema in the AP and HTGP groups, with a higher pancreatic histological score in the HTGP group than that in the AP group (Fig. 2F). Additionally, Lip-1 was found to attenuate pancreatic injury in both AP and HTGP groups. In summary, our rat model was successfully established, and Lip-1 effectively reversed pancreatic injury in this model.

### Verification of ferroptosis in rat model and alleviation by Lip-1 in rat pancreas

Lip-1, proven to be an effective inhibitor of ferroptosis for its strong antioxidative properties^10^, was administered to rats before the induction of AP and HTGP models.

**Figure 3:**
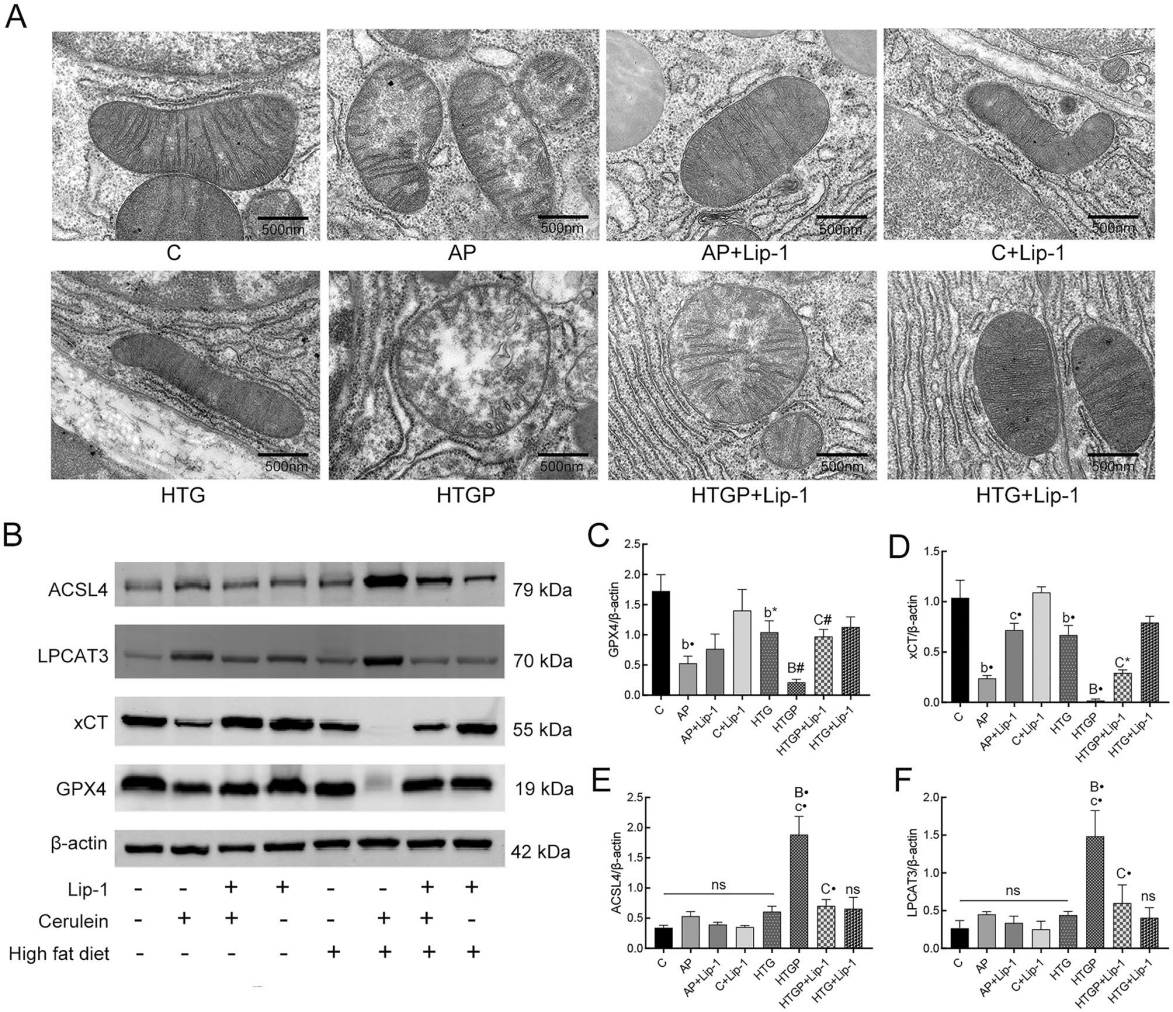
Ferroptosis was observed and alleviated in the pancreas of rats (*n* = 6). (**A**) Ultrastructure of the mitochondria in the pancreas obtained by TEM (×20,000); (**B**–**F**) Western blot analysis of ferroptosis-related proteins, GPX4, xCT, ACSL4, and LPCAT3. GPX4, glutathione peroxidase 4; xCT, cysteine/glutamate transporter; ACSL4, acyl-CoA synthetase long-chain family member 4; LPCAT3, lysophosphatidylcholine acyltransferase 3; C, control; AP, acute pancreatitis; HTG, hypertriglyceridemic; HTGP, HTG pancreatitis; *b vs. the C group, *c vs. the AP group, *B vs. the HTG group, *C vs. the HTGP group, *P < 0.05, ^#^*P* < 0.01, •*P* < 0.001, ns: no significance.

Ultrastructural images of the pancreas were examined to determine if ferroptosis was induced in our models. TEM of mitochondria (Fig. 3A) revealed morphological features such as shrunken mitochondria and reduced mitochondrial crista, indicative of ferroptosis in the AP and HTGP groups. These changes were alleviated with the administration of Lip-1. The levels of core proteins related to ferroptosis were further investigated (Fig. 3B–F). The expression of GPX4 and xCT was downregulated in AP and HTGP groups compared to that in the C and HTG groups (Fig. 3C,D). Additionally, ACSL4 and LPCAT3, proteins involved in the lipometabolic process, were positively upregulated in the HTGP group compared to that in the C and HTG groups(Fig. 3E,F). Lip-1 administration led to the recovery of GPX4, xCT, ACSL4 and LPCAT3 in the HTGP + Lip-1 model. In summary, ferroptosis was induced by HTG and cerulein in our study, and Lip-1 ameliorated pancreatitis-induced injury by targeting lipometabolism-mediated ferroptosis in HTGP.

### The ferroptosis-associated protein level in the pancreas established by HTG and cerulein

**Figure 4:**
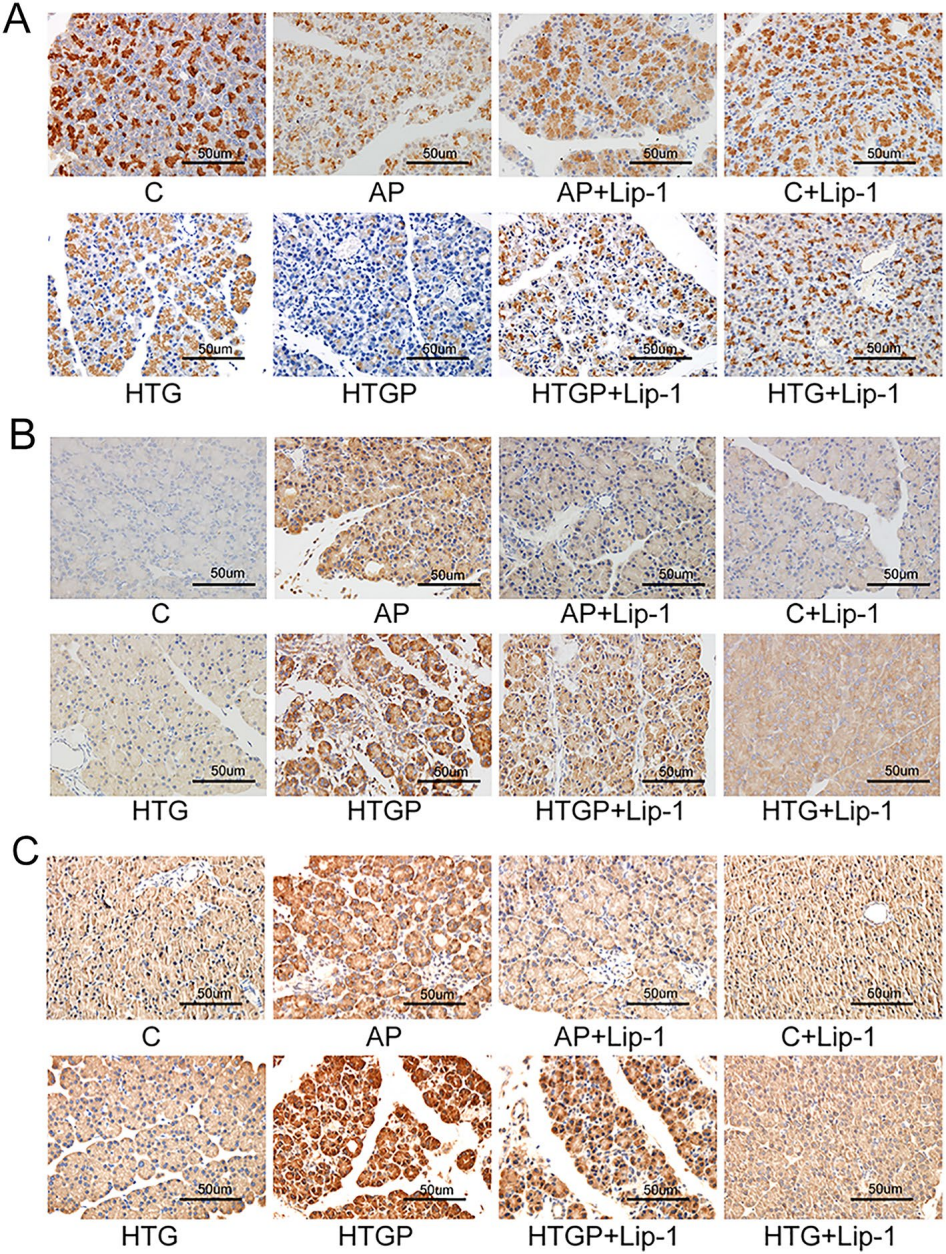
Immunohistochemically stained pancreas tissue (*n* = 6). Protein levels of (**A**) GPX4, (**B**) ACSL4, and (**C**) LPCAT3 in rats (×400). GPX4, glutathione peroxidase 4; xCT, cysteine/glutamate transporter; ACSL4, acyl-CoA synthetase long-chain family member 4.

To explore the protein levels associated with ferroptosis, IHC staining on pancreas specimens was performed. The level of GPX4 was notably downregulated the in the AP group, particularly in HTGP models (Fig. 4A), compared to that in the C group and HTG groups. Administration of Lip-1 in the AP + Lip-1 and HTGP + Lip-1 groups generally ameliorated the suppression of GPX4. Similarly, ACSL4 (Fig. 4B) and LPCAT3 (Fig. 4C) were upregulated in the AP and HTGP groups, consistent with our previous Western blot analysis. Based on these results, lipid metabolism appears to be an indispensable process linked to ferroptosis, and the robust antioxidant properties of Lip-1 can reduce pancreatitis-induced injury, especially in HTGP.

### Lip-1 ameliorates ROS accumulation and alleviates biochemical markers targeting ferroptosis

**Figure 5:**
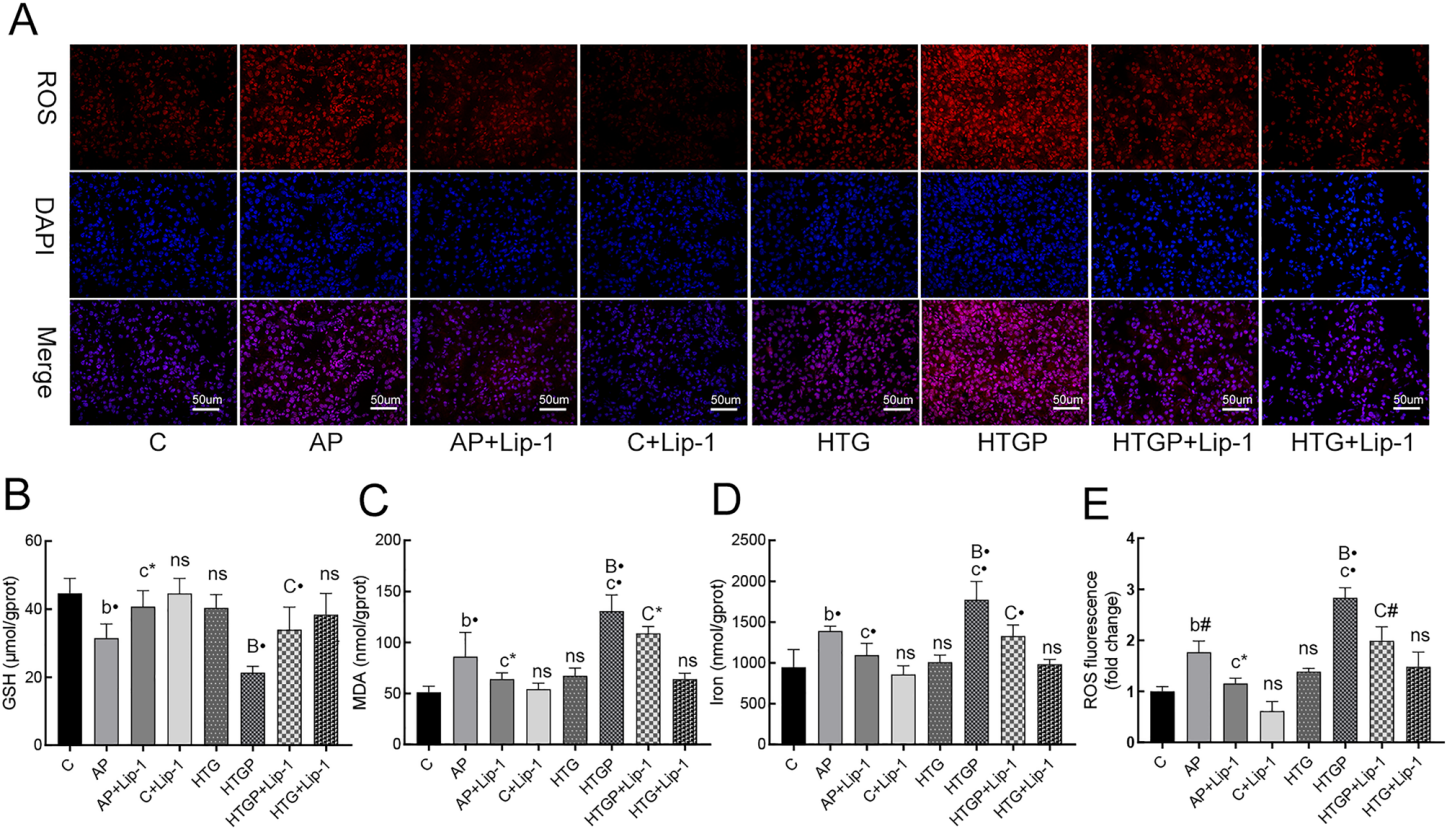
Pancreatic tissues were obtained to detect ROS level, MDA, GSH, and iron content in rats (*n* = 6). (**A**) Representative images of fluorescence probe for ROS (×400); (**B**) Level of GSH in pancreatic tissue; (**C**) Iron content in pancreatic tissue; (**D**) Level of MDA in pancreatic tissue; (**E**) Semi-qualification analysis of ROS in the pancreas of the groups. ROS, reactive oxygen species; MDA, malondialdehyde; GSH, glutathione; C, control; AP, acute pancreatitis; HTG, hypertriglyceridemic; HTGP, HTG pancreatitis; *b vs. the C group, *c vs. the AP group, *B vs. the HTG group, *C vs. the HTGP group, *P < 0.05, ^#^*P* < 0.01, •*P* < 0.001.

The depletion of GSH, drastic lipid peroxidation, and the generation of ROS are widely accepted as contributors to the ferroptosis process^25^. Pancreatic measurements of ROS, MDA, and GSH revealed GSH depletion (Fig. 5B), MDA elevation (Fig. 5C), and uncontrolled ROS generation (Fig. 5A,E) in the AP and HTGP groups. Interestingly, HTGP exhibited a more pronounced accumulation of ROS than the AP group, consistent with increased MDA and decreased GSH. Notably, Lip-1 significantly recovered the levels of GSH, MDA, and ROS. Iron, a pivotal element in metabolism and ferroptosis, was also measured (Fig. 5D). In comparison to that in the C and HTG groups, iron content was increased in the AP and HTGP groups, with HTGP showing a more significant iron overload. However, Lip-1 administration in AP + Lip-1 and HTGP + Lip-1 groups significantly decreased iron overload. In summary, Lip-1 alleviated the biomarkers associated with ferroptosis in the AP + Lip-1 and HTGP + Lip-1 groups.

### ERS participation in ferroptosis induced by HTG and cerulein in the pancreas

**Figure 6:**
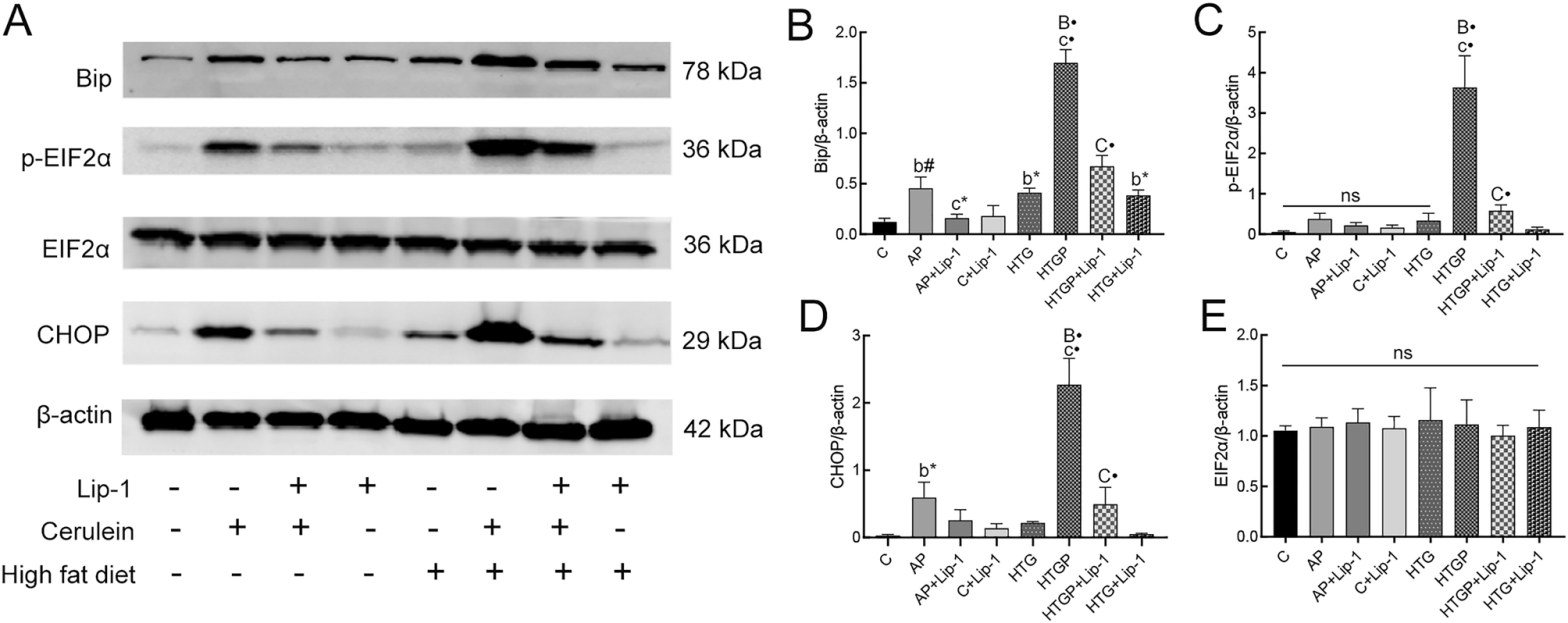
ERS participated in ferroptosis-related pancreatic injury of rats (*n* = 6). Western blot image and analysis of Bip, CHOP, EIF2α, and p-EIF2α in pancreatic tissues (**A**–**E**). C, control; AP, acute pancreatitis; HTG, hypertriglyceridemic; HTGP, HTG pancreatitis; Bip, binding immunoglobulin protein; CHOP, C-EBP homologous protein; EIF2α, eukaryotic initiation factor 2α; p-EIF2α, phosphorylation of EIF2α. *b vs. the C group, *c vs. the AP group, *B vs. the HTG group, *C vs. the HTGP group, *P < 0.05, ^#^*P* < 0.01, •*P* < 0.001, ns: no significance.

Our study confirms the induction of ferroptosis in AP and HTGP models. Recent research indicates that ERS plays a crucial role in promoting pancreatitis^26^, contributing to lipid deregulation^27^. In the present study, the activity of a series of proteins, including Bip, CHOP, EIF2α, and p-EIF2α were assessed in rat pancreas (Fig. 6A). Bip and CHOP were significantly upregulated in the AP and HTGP groups, with HTGP exhibiting a remarkable elevation (Fig. 6B–D), compared to C and HTG groups. Notably, p-EIF2α was significantly higher in HTGP group than that in the AP group. Lip-1 administration in the AP + Lip-1 and HTGP + Lip-1 groups depressed ERS-related proteins, especially in contrast to the HTGP group. EIF2α level showed no significant difference among the groups (Fig. 6E) (*P* > 0.05). These results suggest that ERS participates in AP and HTGP via the Bip/p-EIF2α/CHOP pathway, and its level is downregulated with Lip-1 treatment in the AP + Lip-1 and HTGP + Lip-1 models.

In conclusion, the data indicates that ERS is induced in AP and HTGP models and is alleviated by Lip-1 in vivo.

## Discussion

In the present study, we initially explored ferroptosis in pancreas injury induced by HTGP and compared it with non-HTG-induced AP. Furthermore, we investigated the involvement of ACSL4 levels in ferroptosis-induced AP and HTGP in vivo. For the first time, we concurrently studied lipid metabolism-related proteins, including ACSL4 and LPCAT3, in our rat models. Additionally, our findings confirmed that Lip-1 treatment can effectively alleviate pancreatic injury through the Bip/p-EIF2α/CHOP pathway, particularly in the HTGP group.

Most animals can be induced AP, such as rats, mice, rabbits, et al^28^. SD rats have been widely accepted as subjects of AP since 1952^29^, and most patients with HTGP were male^30^. Besides, our team has rich experience in using male SD rats to establish AP or HTGP models in vivo^14,24,31–33^. Hence, male SD rats were used to establish HTGP model in the study.

AP is characterized by inflammatory-mediated pancreatic injury, a common trigger for systemic inflammatory response and multiple organ failure. Elevated serum triglyceride was independently associated with persistent organ failure in AP^30^. It is widely accepted that a high fat diet can induce HTG models succufully^14,28^. In the present study, the level of serum triglyceride in the non-high fat diet group was much lower than that in the high fat diet group (0.26 ± 0.04 vs. 1.39 ± 0.37, *P* < 0.001), which was in accordance with our previous studies^14,24^. Compared with the AP group, levels of IL-6 and TNF-α were obviously higher in the HTGP group. Additionally, the histological changes in pancreas of the HTGP group was more severe than that in the AP group. These results confirmed that HTGP model was established successfully by a high fat diet and cerulein.

Previous studies have demonstrated the involvement of ferroptosis in the progression of AP both in vivo and in vitro^16,34^. Moreover, several studies have shown that treatment with ferrostatin-1 can suppress ferroptosis-related injuries such as lipid oxidation and oxidative stress, along with an upregulation of GPX4^34,35^. Current research also reveals that severe AP induces renal failure, intestinal barrier injury, and lung injury, which are all closely associated with ferroptosis^11,36,37^. Organ dysfunction progression can be mitigated by typical antioxidants like ferrostatin-1, which reduces iron-dependent lipid peroxidation. Interestingly, a recent study reported that ferroptosis plays a significant role in HTGP rather than in AP, as indicated by proteome sequencing analysis^38^. While numerous studies focus on the therapeutic role of targeting ferroptosis in various diseases, including cancer and inflammation^10,20^, the involvement of ferroptosis in HTGP prognosis remains unclear.

Iron overload and lipid peroxidation are core components of ferroptosis. Excessive iron can trigger uncontrolled ROS generation through the Fenton reaction or enzymes containing iron (e.g., lipoxygenase, downstream of ACSL4 and LPCAT3)^10^. Lipid metabolism is a vital aspect of ferroptosis, with ACSL4 and LPCAT3 activating the membrane phospholipid synthesis process initiated by PUFAs. This process leads to the amplification of lipid peroxidation during ferroptosis^8^. Knockout of *ACSL4* in mice has been shown to confer resistance to ferroptosis-induced acute kidney injury^39^, whereas overexpression of *ACSL4* can restore sensitivity to ferroptosis in cells^40^. Inhibition of LPCAT3, either pharmacologically or genetically, protects cells against ferroptosis by disrupting phospholipid membrane synthesis from PUFAs^41^. Moreover, the knockout of *LPCAT3* in mice may lead to lower body weight and PUFAs, through the regulation of lipids absorption^42^. In the current study, our results observed abnormal upregulated of ACSL4 and LPCAT3 in the pancreas of HTGP. This indicates a newly strengthened connection between lipid metabolism and ferroptosis, with HTG potentially amplifying ferroptosis in vivo, consistent with previous study conclusions^38^. Moreover, lipid metabolism proteins, including ACSL4 and LPCAT3, act as contributors during ferroptosis in HTGP. Interestingly, both ACSL4 and LPCAT3 participate in ferroptosis-related lipid metabolism, and their roles are significant.

The induction of ferroptosis can occur through intrinsic and extrinsic ways, thus targeting GPX4 or xCT can be a strategy to induce or inhibit ferroptosis^10^. GPX4 and xCT play a vital role in ferroptosis, acting as a defense mechanism to maintain homeostasis by synthesizing GSH and suppressing lipid peroxidation. Products of lipid peroxidation, such as MDA, amplify the ROS signal, potentially leading to pancreas injury when cerulein is injected^43^. A previous study has associated pancreatic injury with ferroptosis, demonstrating elevated levels of MDA and ROS alongside a decrease in GSH ^43^. Notably, the knockdown of *GPX4* can suppress cell proliferation through ferroptosis in nasopharyngeal carcinoma^44^. In addition, Dai et al. demonstrated that GPX4 depletion in mice can promote experimental pancreatitis and contribute to tumor pathogenesis^45^. Overall, the down-regulated level of GPX4 is considered a biomarker of ferroptosis. Our study reveals a significant decrease in GPX4 and xCT levels in HTGP compared to other groups.

The ER dysfunction is widely linked to inflammation^46^, with previous studies reporting its involvement in ferroptosis-induced colonic epithelial cell death in ulcerative colitis^47^. Recent research by He et al. suggests that ERS can be triggered by pancreatic toxins, leading to the activation of inflammation in the pancreas^48^. Accumulating evidence indicates that ERS can be induced in AP both in vivo and in vitro^49^. ERS is known to promote the generation of the unfolded protein response, aggravating the inflammatory response and resulting in cell death in AP^50^. Additionally, obesity, characterized by chronic inflammation and cellular stress, has been associated with ERS^51,52^.

This study evaluated representative protein levels of Bip, CHOP, EIF2α, and p-EIF2α related to ERS in the pancreas of rat models. Consistently, our results revealed that ERS is induced in both AP and HTGP models via the Bip/p-EIF2α/CHOP pathway for the first time. Lip-1 treatment resulted in the supression of ERS related proteins, including Bip, p-EIF2α, and CHOP. Importantly, our study provides initial evidence supporting the association between ferroptosis and ERS, indicating that ERS-related proteins can be downregulated by Lip-1.

Various anti-ferroptotic agents, such as ferrostatin-1, Lip-1, and β-Carotene, are recognized as ferroptotic inhibitors and widely utilized in vivo and in vitro^10^. In our research, Lip-1 was administered to rats before the induction of AP and HTGP models. The results substantiated the involvement of ferroptosis in the progression of both AP and HTGP, with Lip-1 effectively mitigating AP, particularly in the context of HTGP, by inhibiting ferroptosis execution. Lip-1 treatment demonstrated protective effects against iron overload and ROS generation, reducing inflammation morphologically in both AP and HTGP models. Moreover, our study suggests that Lip-1 can decrease the levels of MDA and iron content in the pancreas of rats. Based on these findings, Lip-1 not only suppressed lipid metabolism-related proteins, ACSL4 and LPCAT3, but also restored the levels of GPX4 and xCT, reduced inflammation factors IL-6 and TNF-α, and alleviated ERS-related proteins, contributing to the mitigation of ferroptosis-induced pancreatitis, prominently in HTGP.

Although our study shares similarities with the previous research carried out by Meng YT et al.^38^, our focus is esspecially on exploring ferroptosis in HTGP from various aspects. In our study, we established the HTG rat model through high fat feeding, rather than P-407. We use TEM to observe ferroptotic changes of mitochondria in the pancreas. Western blotting was used to evaluate levels of GPX4, xCT, ACSL4, and LPCAT3 in the pancreas associated with ferroptosis. IHC staining methods were performed to observe the expression of ACSL4, LPCAT3, and GPX4 in pancreas. Moreover, we conducted in-depth analyses of iron overload, lipid peroxidation, and biochemical markers associated with ferroptosis. Our findings revealed a close relationship between lipid metabolism and ferroptosis, with ACSL4 and LPCAT3 for the first time observed to be significantly upregulated in HTGP, rather than AP. These results showed the pivotal role of lipid metabolism in the pathogenesis of HTGP. Furthermore, our study discussed ERS-related proteins. Notably, we found Lip-1 may alleviate pancreatic injury through ERS.

As a recently discovered cell death pathway, ferroptosis has been extensively studied in various diseases. With the emergence of HTGP, ferroptosis may interconnect with other forms of cell death, including autophagy, pyroptosis, necrosis, apoptosis, etc^34,35,53^. Notably, recent studies have indicated that copper chelators can reduce the sensitivity of ferroptosis^54^, highlighting the complexity of ferroptosis and its association with multiple metal stress. Our study delves into how lipometabolism participates in ferroptosis in HTGP, providing a novel perspective for the development of future HTGP treatment strategies.

However, considering the intricate pathogenesis of pancreatitis, our research addresses one potential mechanism associated with ERS in HTGP. Due to the multifaceted nature of HTGP, there are limitations in our study, primarily focused on a rat model, and further comprehensive evidence is needed to substantiate these findings. More profound prospective research, particularly clinical trials, is essential to confirm the therapeutic effects of ferroptosis in HTGP. In the long run, we believe ferroptosis could emerge as a potent therapeutic target for HTGP.

## Conclusion

Taken together, we conducted a comprehensive comparison of the severity between AP and HTGP. We examined factors such as iron overload, proteins associated with ferroptosis, and ERS, including lipid peroxidation related to ferroptosis. The results suggest that ferroptosis occurred in both AP and HTGP models. Furthermore, our findings indicate that lipid metabolism can exacerbate the severity of pancreatitis by targeting ferroptosis, particularly in HTGP models. Additionally, ERS may participate in ferroptosis via the Bip/p-EIF2α/CHOP pathway, with Lip-1 exhibiting a mitigating effect in the HTGP rat model (Supplementary Information).

### Supplementary Information

Supplementary Information.

## Data Availability

The datasets used and/or analysed during the current study are available from the corresponding author on reasonable request.
