# Supplementary material for: Liproxstatin-1 attenuates acute hypertriglyceridemic pancreatitis through inhibiting ferroptosis in rats
Source: Sci Rep. 2024 Apr 25;14:9548. doi: 10.1038/s41598-024-60159-7 (PMC11045844; doi:10.1038/s41598-024-60159-7)

All replicates of western blots

Figure 3B

ACSL4

Shown in Figure 3B

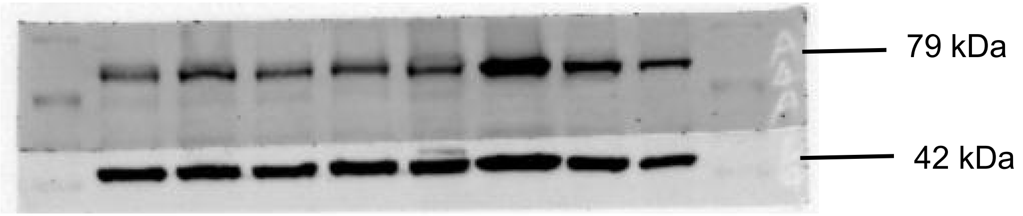

Replicate blot

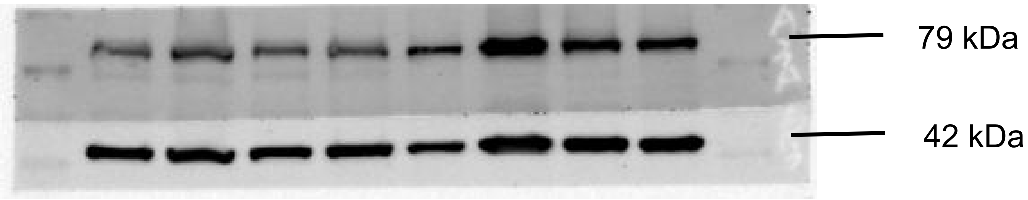

Replicate blot

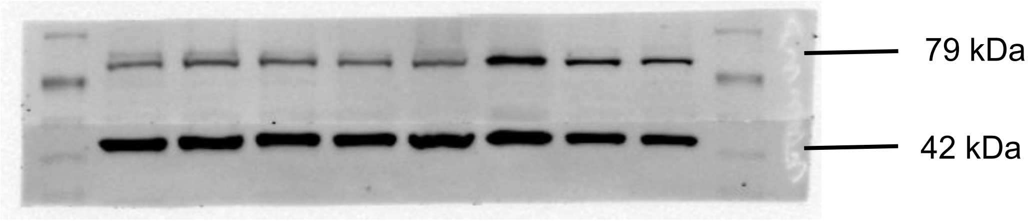

LPCAT3

Shown in Figure 3B

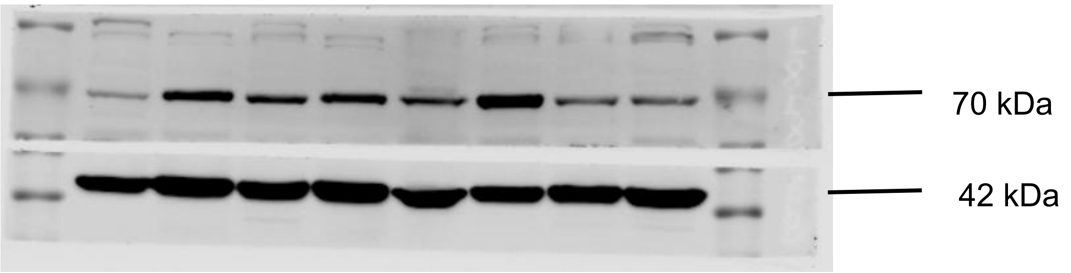

Replicate blot

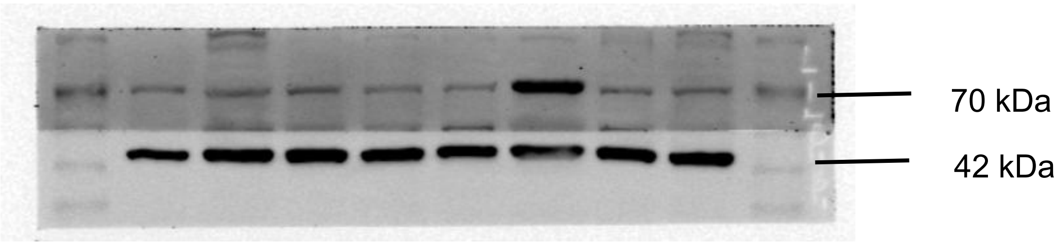

Replicate blot

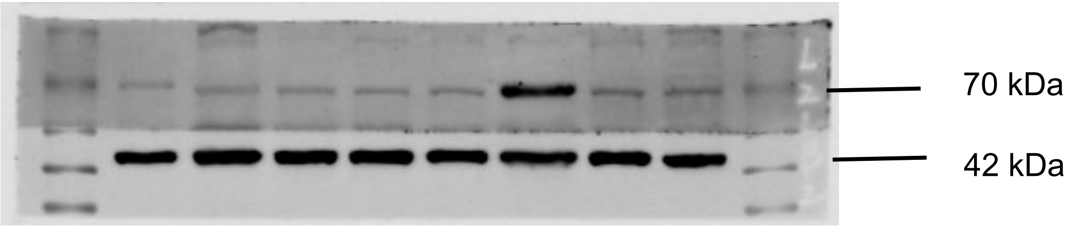

xCT

Shown in Figure 3B

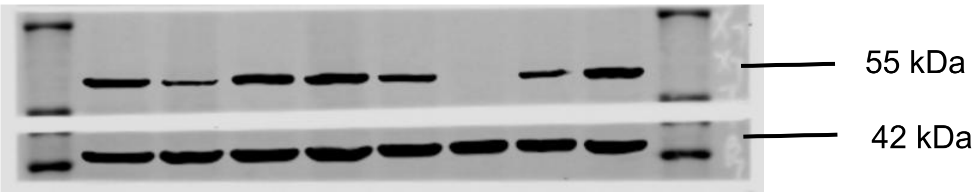

Replicate blot

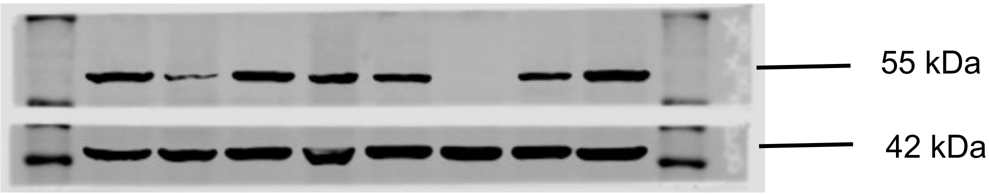

Replicate blot

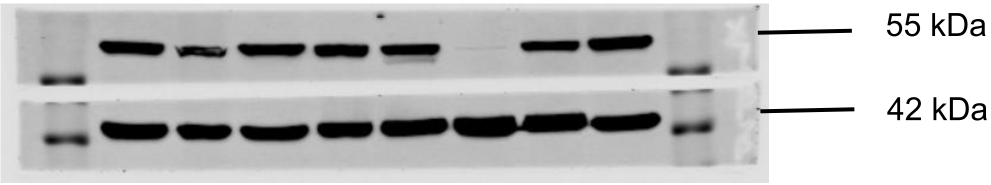

GPX4

Shown in Figure 3B

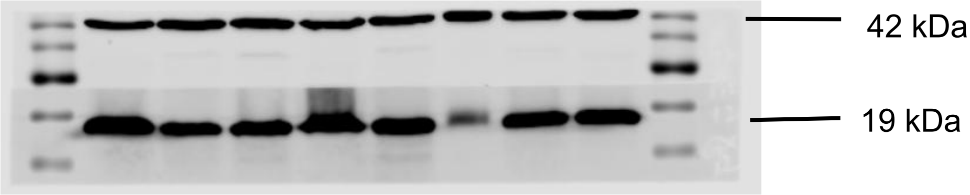

42 kDa

19 kDa

Replicate blot

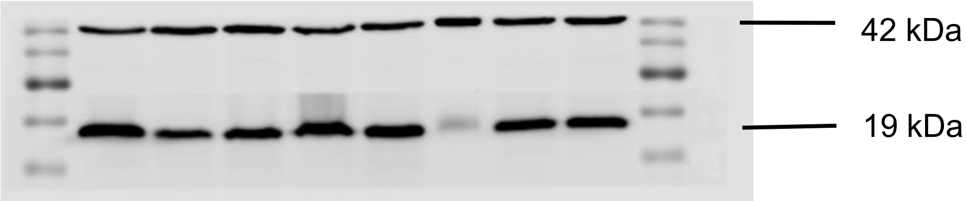

42 kDa

19 kDa

Replicate blot

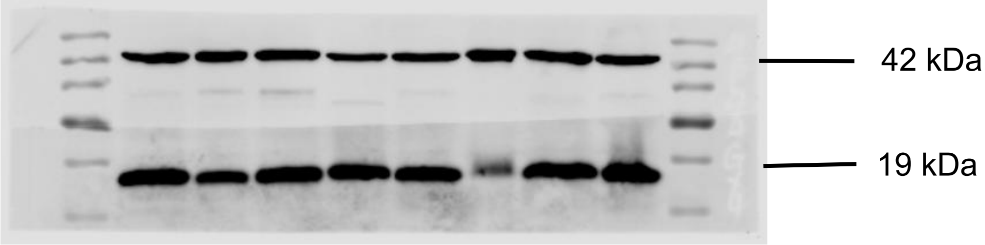

42 kDa

19 kDa

Bip

Shown in Figure 6B

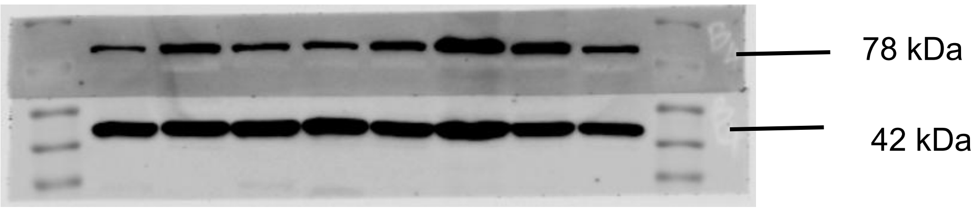

Replicate blot

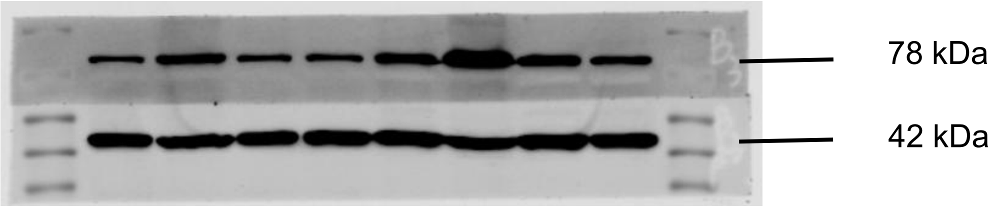

Replicate blot

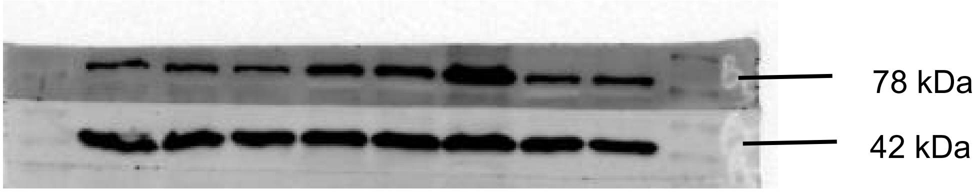

p-EIF2α

Shown in Figure 6B

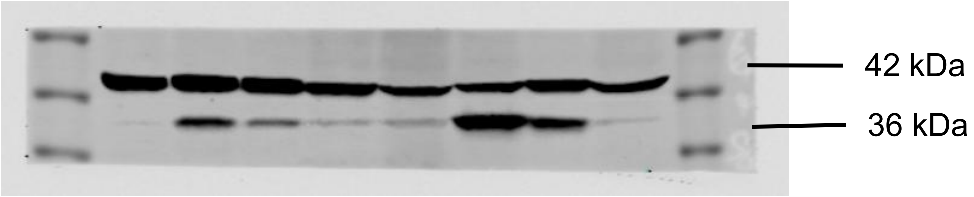

Replicate blot

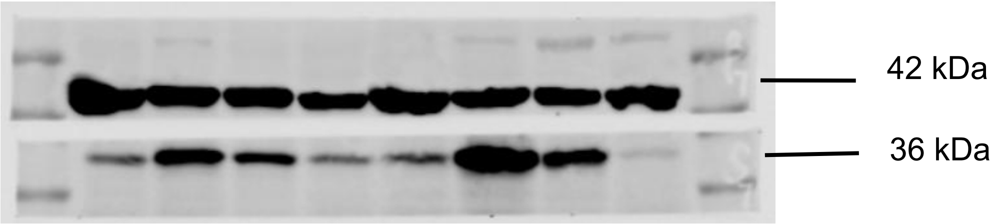

Replicate blot

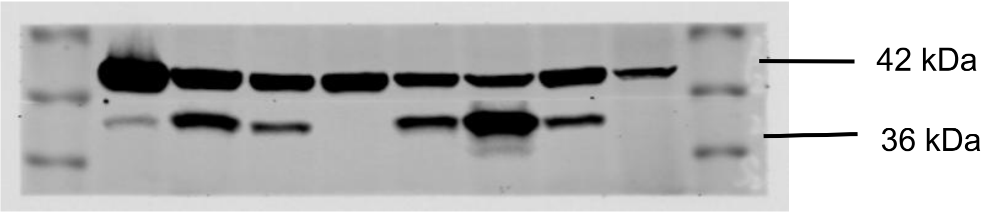

EIF2α

Shown in Figure 6B

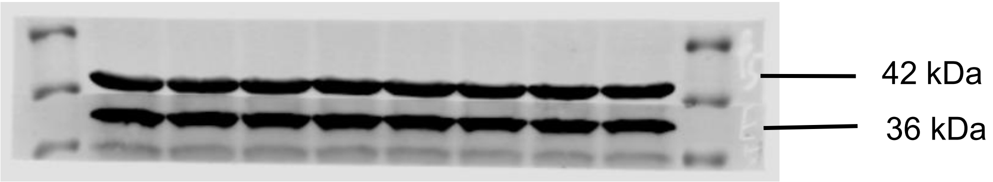

Replicate blot

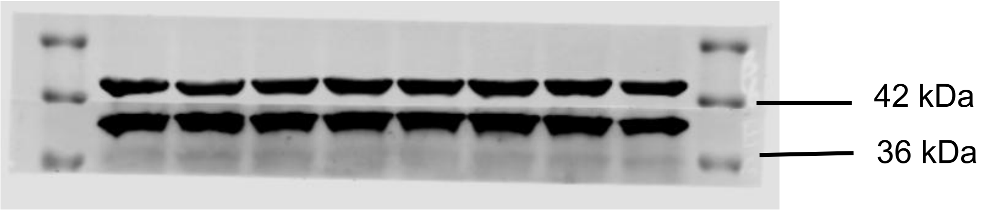

Replicate blot

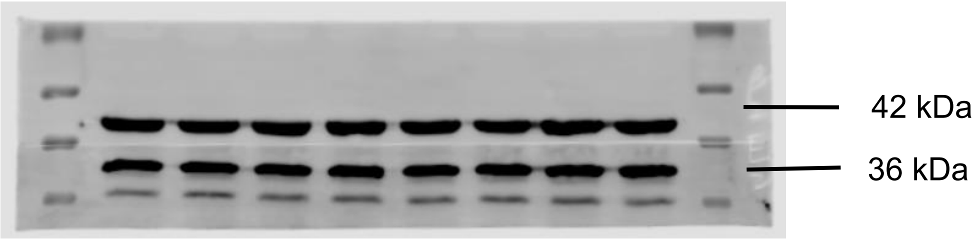

CHOP

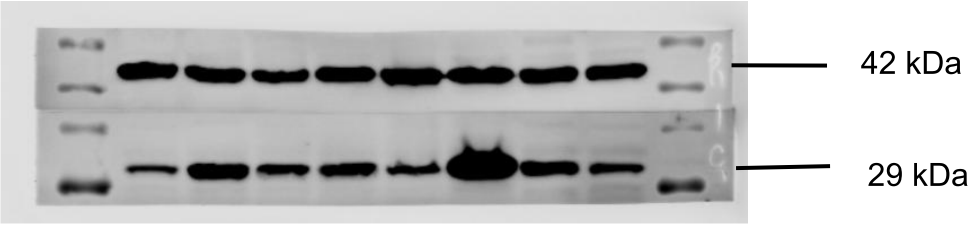

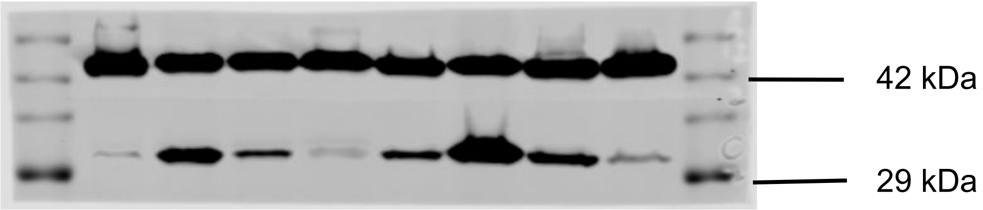

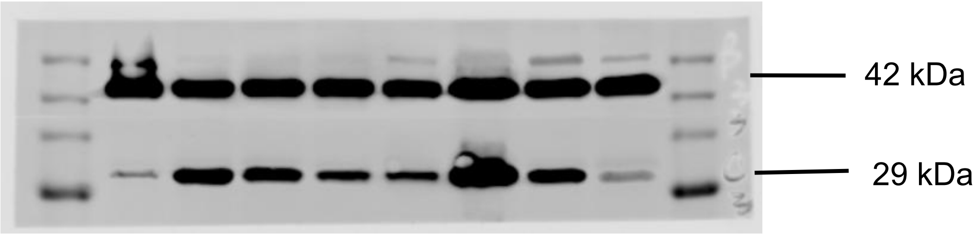

Supplement: Supplementary file 1 — Supplementary Information. [file 41598_2024_60159_MOESM1_ESM.pdf]
